# The Influence of Family Bonding, Support, Engagement in Healthcare, on PrEP Stigma among Young Black and Latino Men Who Have Sex with Men: A Path Analysis

**DOI:** 10.3390/children9030330

**Published:** 2022-03-01

**Authors:** Donte T. Boyd, Gamji M’Rabiu Abubakari, DeAnne Turner, S. Raquel Ramos, Mandy J. Hill, LaRon E. Nelson

**Affiliations:** 1 College of Social Work, The Ohio State University, Columbus, OH 43210, USA; 2 Center for Interdisciplinary Research on AIDS, Yale University, New Haven, CT 06510, USA; mohammed-rabiu.abubakari@yale.edu (G.M.A.); dturner@usf.edu (D.T.); 3 School of Public Health, Yale University, New Haven, CT 06510, USA; 4 College of Nursing, University of South Florida, Tampa, FL 33612, USA; 5 School of Nursing, Yale University, New Haven, CT 06477, USA; raquel.ramos@yale.edu (S.R.R.); laron.nelson@yale.edu (L.E.N.); 6 McGovern Medical School, University of Texas Health Science Center at Houston, Houston, TX 77030, USA; mandy.j.hill@uth.tmc.edu

**Keywords:** HIV, PrEP, adolescents, families, stigma, MSM

## Abstract

This study employs the ecodevelopmental theory to examine the influence of mother and father bonding, family engagement in healthcare, and family support on PrEP stigma among BLMSM. We used a cross-sectional sample from wave five of the Healthy Young Men (HYM) study, with a survey sample of 399 participants aged 16–24 years. We conducted two-path analyses to test multiple hypotheses: (1) mother/father bonding is associated with an increase in family engagement in healthcare; (2) family engagement in healthcare is associated with family social support; and (3) family social support is associated with PrEP stigma. Family social support was negatively correlated with PrEP stigma (r = −0.15; *p* < 0.001). The findings show that families either led by a Black/Latino father or mother have a significant impact on the sexual health-seeking behavior of BLMSM and their perception of HIV and PrEP.

## 1. Introduction

Although pre-exposure prophylaxis (PrEP) proves efficacious in reducing HIV transmission, not all populations benefit from the advancement equally [1,2]. Black and Latino men who have sex with men (BLMSM) have lower rates of access and uptake of PrEP compared with their white counterparts [3,4,5,6]. Among MSM qualifying for a PrEP prescription in 2019, Black and Latino men showed 8% and 14% prescription rates, respectively, compared with white men at 63% [7]. Meanwhile, BLMSM continue to be disproportionately infected by HIV, with Black and Latino MSM representing 37% and 30% of MSM with new HIV diagnoses in 2018, respectively, compared with white MSM of 27% [8].

Social and structural factors have impacted PrEP uptake among BLMSM. These factors are structural health care barriers, such as lack of access, insurance status, and discrimination/racism. Social factors emanating from homophobia, HIV stigma, and additional personal elements within the cultural/community context have also contributed to low PrEP use [5,6,9,10,11]. Stigma continues to create challenges to PrEP uptake. Stigma is the social process of ascribing “negative” perceptions or disapproval to a specific individual or personal attribute. Stigma could result in actions such as discrimination against persons with such ”spoilt” identities or supposed “negative” qualities [5,12]. In certain Black and Latino communities, the notion that PrEP is meant exclusively for gay men reduces the interest of PrEP for BLMSM if they are concealing their sexual identity or disassociating from homosexuality [5,10]. Sexual orientation concealment and/or disassociation from homosexuality in many cases are due to fear of negative consequences within the family unit. Improvement in family relationships and parental bonding may extend the reach of PrEP among adolescents [13,14].

Parental bonding is a subjective experience of affection from a parent towards a child. The core tenet of the bond is the perceived feeling of “love” expressed in parental behavior [15,16,17]. There is a dearth of literature on parental bonding in BLMSM and its effects on their PrEP attitudes, stigma, and use and perceptions of stigma. However, previous studies associate child–parent communication to current PrEP use and the perceived lack of parental support of PrEP use to low interest in initiating PrEP [14,15,16,17]. Parental bonding or parental communication may improve many sexual health outcomes, such as the age of sexual debut, number of sexual partners, reduction in HIV risk behaviors, and the acquisition of other sexually transmitted infections [15,16,17]. For example, adolescents who talk to their parents about condoms are more likely to use them [18]. However, findings show some inconsistency in the role of family and HIV prevention behaviors with mother support positively predicting condom use compared with a negative prediction by father bonding [15,16,17,19]. Hence, more research is needed to understand the role of parental bonding and other familial factors in reducing HIV and PrEP-related stigma.

To address the gaps in knowledge surrounding this area, this study used the ecodevelopmental theory to examine how mother and father bonding, family engagement in healthcare, and family support influence PrEP stigma among BLMSM and fills a critical gap in the literature on the same. We hypothesized the following:

**Hypothesis** **1** **(H1).** *Mother and Father Bonding will predict Family Sexual Health.*

**Hypothesis** **2** **(H2).** *Family Sexual Health will predict Family Social Support.*

**Hypothesis** **3** **(H3).** *Family Social Support will predict PrEP Stigma.*

### Ecodevelopmental Theory

The ecodevelopmental theory guided this study to investigate how the family context (i.e., mother bonding, father bonding, and family engagement in healthcare) influences PrEP stigma among BLMSM. Based on Bronfenbrenner’s social-ecological model, the theory frames the social ecology of an individual in the context of four interrelated systems: microsystem, mesosystem, exosystem, and macrosystem [20,21]. To date, the ecodevelopmental theory has been useful in framing the influence of family factors on HIV attitudes, HIV testing, and social support [15,16,17]. Furthermore, the ecodevelopmental theory stresses how family functioning and interactions within the family contribute to youth risk or serve as a protective mechanism through a developmental lens [22].

In understanding how stigma influences HIV prevention behaviors such as PrEP, it is critical to recognize the intersecting identities of BLMSM and the potential operation of these identities in the family context. Intersectionality can complement and enhance the ecodevelopment theory, as intersecting stigmas may be critical drivers of PrEP-related stigma among BLMSM [23]. While both ecodevelopment theory and intersectionality research enhance our understanding of oppression and disparities in public health and the family context, these theoretical frameworks can jointly highlight the importance of family and health and how to reduce stigma around PrEP and HIV.

## 2. Methods

### 2.1. Procedures

This secondary analysis used wave five data from the Healthy Young Men’s (HYM) cohort study, a longitudinal study conducted with a sample (*n* = 498) of MSM of color. The HYM research aims to prevent and reduce HIV acquisition among MSM of color by investigating barriers and protective factors that contribute to their engagement in care. Young men who were HIV negative (*n* = 448) and positive (*n* = 50) were eligible to participate in the study. Inclusion criteria were: (1) between the ages of 16 to 24 years; (2) assigned as male at birth; (3) self-identified as gay, bisexual, or uncertain about their sexual orientation; (4) reported a sexual experience with a man within the last 12 months; (5) self-identified as of African American/Black, Hispanic/Latino, or multiracial ethnicity; and (6) resided in Los Angeles city or county with no plans on moving for at least six months.

Described in a meticulous manner [24], the study procedures included both venue (e.g., bars, coffee shops, and parks) and social media-based (e.g., Facebook, Instagram, and Grindr). Participants were recruited in Los Angeles, California, and the surrounding cities/counties. There were 1371 people screened for the study, and among those, 40% (550) were eligible to participate. Respondents were asked to participate in data collection at baseline and follow-up every 6 months. Participants were asked to contact their interviewer monthly (e.g., text message, phone call, or Snapchat). In return, they would receive a USD 7 monthly incentive (the additional USD 42 added to their data collection incentive) [24]. They were provided written informed consent during a face-to-face recruitment event, and each person received USD 65 for their study visit. This study received Institutional Review Board approval from the Children’s Hospital, Los Angeles.

### 2.2. Measures

The outcome variable for this study was PrEP stigma and was measured by an 11-item, 5-point Likert scale, with values ranging from 1 (strongly disagree) to 5 (strongly agree), with a higher score indicating lower stigma (α = 0.84). Sample items provided here were in response to the prompt, stating, “Please decide how much you agree with the following statements: (1) “I think people should take PrEP.” (2) “Having sex with someone on PrEP is risky”, and (3) “People have different opinions about PrEP.”

PrEP attitudes were measured by a 3-item, 5-point Likert scale with values ranging from 1 (strongly disagree) to 5 (strongly agree), with higher scores reflecting positive attitudes towards PrEP (α = 0.83). A sample item stated, “I think people should take PrEP” [25].

Family social support was measured using a 4-item Likert scale (ranging from 1 (strongly disagree) to 4 (agree)), asking the respondents to agree or disagree with the following: (1) “My family tries to help”, (2) “I can talk about my problems with my family”, (3) “My family is willing to help me make decisions”, and (4) “I get the emotional help and support I need from family”. Family Social Support (FSS) scores were created by averages across item responses (α = 0.91). Mother and father bonding were two separate measures created using a 2-item scale (ranging from 0 = no, 1 = yes), asking about participants’ receiving love and support from their mother and father. Sample items included (1) “Would you say this woman/man was loving?” and (2) “Would you say this woman/man was very supportive?”. The scores were achieved by averaging across item responses, and the Cronbach’s alpha for fathers was α = 0.76 and mothers α = 0.80.

Family engagement in healthcare was measured by two items, consisting of 5-point Likert-type questions, with values ranging from 1 (strongly disagree) to 5 (strongly agree) and a higher score indicating more family engagement in healthcare (α = 0.70). A sample item includes “When I was growing up, my parent(s) or guardian(s) made sure I had regular check-ups with my doctor”.

### 2.3. Statistical Analysis Plan

All analyses were conducted on observations inclusive of non-missing data for the outcome of PrEP stigma. Table 1 presents the sample characteristics of Black and Latino MSM (*n* = 498). Table 2 shows the bivariate correlations between the predictors and the outcome variable (PrEP stigma). Table 3 and Table 4 present the standardized and unstandardized results of path analysis. Two path analyses were used to examine the associations between mother and father bonding history, history of family healthcare, family social support, and PrEP stigma. The model fit was considered good if the χ2 value was non-significant, comparative fit index (CFI) 0.95, Tucker–Lewis index (TLI) 0.95, and the root mean square error of approximation (RMSEA) was ≤0.06 (adequate if ≤0.08). The Akaike information criterion (AIC) and Bayesian information criterion (BIC) were utilized to compare the fit between the models. These fit indices were assessed as generated path models. The Bollen–Stine bootstrap procedures with 6000 bootstraps resampled were also used to assess the consistency of the proposed model with the sample data, which was indicated by the results with a *p*-value greater than 0.05. A mean score of the scale items was generated for participants with non-missing data for survey scales. All analyses were conducted using STATA 17.

### 2.4. Sample Characteristics

Table 1 provides sample characteristics of participants in the HYM study. The study sample consisted of 399 MSM color between the ages of 18 to 29. The mean age was 22 during wave 5. Most of the sample self-identified as Latinx (59%), followed by 41% as African American. Seventy-six percent self-identified as gay, 17% bisexual, 4% MSM, 2% pansexual, 1% heterosexual, and approximately 1% unsure or questioning. Additionally, 27% of the participants reported working full time. Nineteen percent reported not working but looking for work. Twenty-six percent of the individuals stated that they use a condom during receptive anal sex all the time, and 28% said that they use a condom as the insertive partner all the time. Most of the sample (86%) reported not using PrEP.

Overall, approximately 50% of BLMSM experienced some form of PrEP stigma. Generally, 72% harbored positive attitudes towards PrEP, and 71% reported having family support. Moreover, only 63% of the sample reported their family being engaged in healthcare. Most of the sample reported having strong bonds with their mothers (89%), and 66% reported strong bonds with their fathers.

### 2.5. Bivariate Correlations

Table 2 presents the bivariate correlations between the criteria and outcome variables. PrEP attitudes were negatively correlated with PrEP stigma (r = −0.41; *p* < 0.001). Family social support was negatively correlated with PrEP stigma (r = −0.15; *p* < 0.001). Family engagement in healthcare was negatively associated with PrEP stigma (r = −0.04; *p* < 0.05) and positively associated with PrEP attitudes (r = 0.03; *p* < 0.05) and family social support (r = 0.21; *p* < 0.001). History of mother bonding was positively associated with family social support (r = 0.34; *p* < 0.001) and family engagement healthcare (r = 0.13; *p* < 0.05). History of father bonding was positively correlated with family social support (r = 0.40; *p* < 0.001) and mother bonding (r = 0.50; *p* < 0.001).

### 2.6. Path Analysis

#### 2.6.1. Mother Bonding

**Figure 1:**
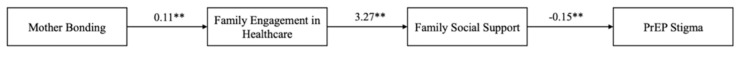
Direct pathways to reduction in PrEP stigma through mother bonding, (*n* = 399). Note: standardized path coefficients presented. ** *p* < 0.01.

**Figure 2:**
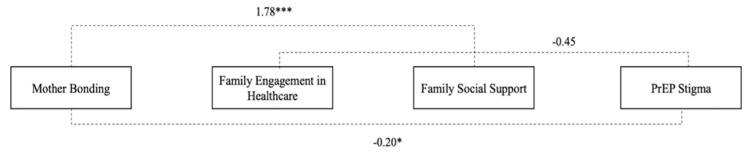
Indirect pathways to reduction in PrEP stigma (*n* = 399) mother bonding. Note: standardized path coefficients presented. * *p* < 0.05; *** *p* < 0.001.

The model demonstrated a good overall model fit for the sample data (χ2 = 71.0 (6), *p* = 0.67; CFI = 0.99; TLI = 0.99; RMSEA = 0.01; AIC = 45; and BIC = 46). Table 3 lists the unstandardized and standardized results for mother bonding (Figure 1) (*n* = 399). Results indicated that a history of mother bonding was statistically significant and directly linked to family engagement in healthcare (β = 0.13; *p* = 0.011). Family engagement in healthcare was significant and directly linked to family social support (β = 2.67; *p* < 0.011). Family social support was significant and negatively associated with PrEP stigma (β = −0.15; *p* < 0.010). Mother bonding was significant and indirectly associated with family social support (β = 1.78; *p* < 0.001). Lastly, mother bonding was significant and indirectly linked with PrEP stigma (β = −0.20; *p* < 0.001) (Figure 2).

#### 2.6.2. Father Bonding

**Figure 3:**
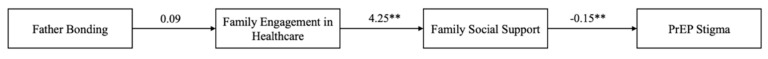
Pathways to PrEP stigma through father bonding (*n* = 245). Note: standardized path coefficients presented. ** *p* < 0.01.

**Figure 4:**
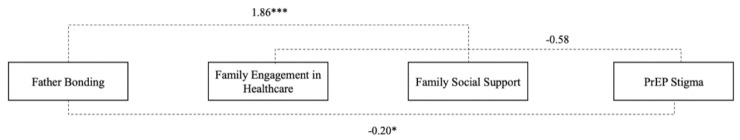
Indirect pathways to reduction in PrEP stigma (*n* = 399) through father bonding. Note: standardized path coefficients presented. * *p* < 0.05; *** *p* < 0.001.

The model demonstrated a good overall fit for the sample data (χ2 = 77.65 (6), *p* = 0.99; CFI = 0.99; TLI = 0.99; RMSEA = 0.01; AIC = 46; and BIC = 47). Table 3 shows the unstandardized and standardized results for father bonding (Figure 3) (*n* = 245). Family engagement in healthcare was significant and directly associated with family social support (β = 4.25; *p* < 0.01). Family social support was significant and negatively linked with PrEP stigma (β = −0.15; *p* < 0.001). Father bonding was significant and indirectly associated family social support (β = 1.86; *p* < 0.001). In addition, father bonding was significant and indirectly associated with PrEP stigma (β =−0.20; *p* < 0.05) (Figure 4).

## 3. Discussion

The purpose of this study was to use the ecodevelopment theory to examine the impact of family-level factors on PrEP stigma among BLMSM. Despite the low PrEP use among BLMSM and the significant public health implications, few studies have examined the contextual factors associated with mother and father bonding, family engagement in healthcare, and family social support on PrEP stigma [13,14,15,16,17], making our study novel and relevant. Overall, our findings highlighted that the influence of the familial microsystem is pronounced in modifying PrEP stigma. Results also indicated that mother and father bonding predicted family engagement in healthcare, which in turn predicted family social support and reduction in PrEP stigma. These findings allow for a more nuanced understanding of the extent to which family-level factors may affect PrEP stigma, which holds implications for the health and well-being of BLMSM.

Studies examining the effects of the ecodevelopmental theory in the family context on BLMSM health, HIV, and stigma are limited [26]. Ecodevelopmental theory postulates that youth are embedded in an interrelated and interconnected context [26], and researchers have focused primarily on the family microsystem. These study findings demonstrate the importance of family factor influences on PrEP stigma among BLMSM. This is significant because our study expands the current state of the science by indicating the importance of the family and its dynamics within a developmental framework. Our research findings suggest that the family context contributes to engagement in healthcare, social support, and reduction in HIV stigma. Additionally, in this study, both mothers and fathers are critical to reducing PrEP stigma. Utilizing the ecodevelopment theory allows further understanding of the role and need of parents and families as support in reducing PrEP stigma and increasing uptake among these populations, as indicated in the three-study hypothesis.

Our study results indicated that mother bonding both positively predicted family engagement in healthcare but not for father bonding (Figure 1), which is consistent with prior literature that parental bonding influences communication, self-esteem, positive health outcomes, and positive attitudes towards HIV prevention behaviors [13,14,15,16,17]. The presence of family constitutes an important source of psychological stability for individuals who need healthcare [27], in this case, BLMSM disproportionately affected by HIV, who can use PrEP. Our results underscore the importance of mothers being involved in their son’s healthcare and understanding their roles in ensuring their children learn the importance of visiting a healthcare provider at early stages in life. Mothers have opportunities to continue to nurture their bond by engaging their sons around sexual health communication, the importance of HIV/STD testing, condom use as a family, or using a healthcare provider. Even though father bonding was not significant with family healthcare engagement, this allows researchers to explore the father’s role in their son’s healthcare and how we can further engage them in this process. This is critical because families who share bonds improve mental health and support while engaging in healthcare and reducing the stress and stigma around certain topics such as HIV.

The path analyses indicated that family engagement in healthcare was associated with family social support. Past research has shown that family engagement and support in care are essential for optimal health outcomes [27]. Additionally, families play a significant role in promoting health and wellness [27], which is critical for BLMSM, who are disproportionately affected by HIV and less likely to use PrEP. Moreover, one study indicated that black males consistently visited the doctor because their fathers always engaged in healthcare [28]. This is a form of socialization and a tangible expression of the Black male influence through role modeling of healthy behaviors, which can help improve the health and well-being of BLMSM [27,29]. BLMSM who have a history of family engagement in healthcare may live in healthy environments. They may have the support needed to understand the importance of routine care [29]. Through understanding the importance of routine care and family social support, they may have positive experiences with their providers who can provide essential and accurate information on HIV testing and PrEP, reducing their stigmatizing views towards PrEP and HIV.

Our study findings indicated that family social support in both path analyses (mother and father) was independently associated with a reduction in PrEP stigma. This is an important finding because PrEP stigma has been linked to lower PrEP interest, intentions, comfort discussing PrEP with a provider, and uptake [10,11,28]. Previous literature has found that higher levels of support increase HIV testing and PrEP use [13,14,15,16,17]. As parents are critical to the well-being of BLMSM, increased attention is needed to family-based HIV prevention, such as efforts to integrate parent–son discussions regarding PrEP, as parents often serve as gatekeepers to biomedical intervention and can function as a support system [28]. Engaging parents in HIV prevention may reduce PrEP stigma. This finding further disproves the perceived belief among the youth that parents will not support the utilization of PrEP. There are mixed findings indicating that father bonding will impede healthcare usage, thus, highlighting that it is essential to bond with parents regardless of their being mothers or fathers.

Surprisingly, our results indicated that mother and father bonding was indirectly associated with reducing PrEP stigma. This is critical as PrEP stigma minimizes the likelihood of engagement in PrEP [10,11]. However, if we can increase parents’ awareness of PrEP and the importance of their sons using PrEP through education, we may reduce their stigmatizing views on HIV and PrEP. This is an opportunity for health educators, researchers, and practitioners to talk with parents about HIV prevention and care as a continuum. It will then help underscore the importance of PrEP and may lead to an overall reduction in stigma around PrEP. As noted in our findings, parental bonding with their sons is the first step in removing stigma.

Although our study findings contribute to the literature on family social support and PrEP stigma, there are several limitations. The analysis was conducted using secondary data, so we were dependent on the pre-existing measures to determine variables related to family context. Additionally, the landscape for the availability and administration of PrEP (as an oral medication or as an injectable) is ever changing. We are unsure how the availability of PrEP may or may not influence its uptake in the future. The data analyzed were cross sectional, and we cannot predict changes in family dynamics, including divorce or single parenthood, from the data. Future studies should examine the longitudinal impact of family bonding and how it changes over time in different household settings. We plan to augment our research findings by examining the differences between household types based on socioeconomic factors and demographics such as single parenthood.

## 4. Conclusions

The purpose of our ecodevelopmental study was to examine family-level factors on PrEP stigma among BLMSM. Based on our findings, there is an urgent need to optimize HIV outcomes by including the family unit in Black and Latino HIV prevention. Utilizing the family unit and providing comprehensive resources to increase HIV prevention awareness may reduce stigma around PrEP and encourage use.

Attention to family dynamics and leveraging the strengths of BLMSM may help reduce PrEP stigma and negative attitudes towards PrEP. Much bio-behavioral research focuses on risk factors associated with PrEP [11]; however, to optimize HIV prevention outcomes for BLMSM, we need to include the family unit as a means of support in HIV prevention. Family-based interventions have been proven efficacious in HIV prevention [13]. Research should consider the benefits that PrEP offers in addition to HIV protection (e.g., reduced HIV anxiety, increased sexual autonomy, and enhanced comfort with serodiscordant relationships), which could help to reshape PrEP messaging and delivery and, more importantly, reduce stigma.

## Figures and Tables

**Table 1:** Sample Characteristics (*n* = 399).

Variable	Frequency (%)
Age, in years (mean, (SD))	22 (2.01)
Race	
Latino	250 (59%)
Black	174 (41%)
Education	
College graduate and above	282 (22%)
Some college/AA	631 (50%)
High school/AA	239 (19%)
9th–12th	36 (3%)
Employment	
I am not working at this time	43 (10%)
Yes, part-time	172 (38%)
Yes, full-time	123 (27%)
Not working at this time and NOT looking	20 (4%)
Not working at this time but looking for work	84 (19%)
Sexual Orientation	
Homosexual (gay or bisexual)	334 (76%)
Heterosexual (straight)	1 (0.22)
Bisexual	74 (17%)
Other same sex (e.g., MSM)	20 (4%)
Pansexual	11 (2%)
Unsure/questioning	4 (0.89%)
Other—please specify	2 (0.45)
Do not know	2 (0.45)
In the last 30 days, how often did you use a condom during ANAL receptive sex?	
0–25% of the time	98 (36%)
26–50% of the time	30 (11%)
51–75% of the time	33 (12%)
76–99% of the time	39 (14%)
100% of the time	70 (26%)
In the last 30 days, how often did you use a condom during ANAL insertive sex?	
0–25% of the time	113 (39%)
26–50% of the time	35 (12%)
51–75% of the time	20 (7%)
76–99% of the time	42 (14%)
100% of the time	82 (28%)
PrEP Use	
Yes	56 (14%)
No	343 (86%)

**Table 2:** Bivariate Correlations on PrEP Stigma (*n* = 399).

PrEP Stigma	1					
PrEP attitudes	−0.41 ***	1				
Family social support	−0.15 ***	0.07	1			
Family engagement in healthcare	−0.04 *	0.03 *	0.21 ***	1		
History of mother bonding	0.01	0.01	0.34 ***	0.13 *	1	
History of father bonding	−0.02	0.06	0.40 ***	0.04	0.50 ***	1
Mean	4	3.62	5	7.01	0.81	0.66
SD	1.1	0.69	1.49	2.2	0.29	0.36
Range	2.0–8.0	2.0–5	1.0–7.0	2.0–11.0	0.0–1.0	0.0–1.0

*p* < 0.05 *, *p* < 0.001 ***.

**Table 3:** Path Analysis Mother Bonding on PrEP Stigma (*n* = 399).

Observed	B	95% CI	SE	β
Direct Effects				
Family engagement in healthcare				
Mother bonding	0.44	0.02, 0.85	0.21	0.11 **
Family social support				
Family engagement in healthcare	4.05	0.29, 7.80	1.91	3.27 **
PrEP stigma				
Family social support	−0.11	−0.18, −0.04	−0.04	−0.15 **
Indirect Effects				
Family social support				
Mother bonding	1.78 ***	1.29, 2.26	0.24	
PrEP stigma				
Family engagement in healthcare	−0.45	−0.96, 0.06	0.26	
Mother bonding	−0.20 *	−0.33, −0.05	0.07	

*p* < 0.05 *, *p* < 0.01 **, *p* < 0.001 ***.

**Table 4:** Path Analysis Father Bonding on PrEP Stigma (*n* = 245).

Observed	B	95% CI	SE	β
Direct Effects				
Family engagement in healthcare				
Father bonding	0.35	10.01, 0.71	0.18	0.09
Family social support				
Family engagement in healthcare	5.27	0.08, 10.47	2.64	4.25 **
PrEP stigma				
Family social support	−0.11	−0.18, −0.04	0.04	−0.15 **
Indirect Effects				
Family social support				
Father bonding	1.86 ***	1.37, 2.34	0.25	
PrEP stigma				
Family engagement in healthcare	−0.58	−1.27, 0.10	0.35	
Father bonding	−0.20 *	−0.35, −0.06	0.07	

*p* < 0.05 *, *p* < 0.01 **, *p* < 0.001 ***.

## Data Availability

Data must be requested from the authors. The present study was conducted using secondary data from the Healthy Young Men Study.
